# Draft Genome Sequence of the Nitrogen-Fixing and Hormogonia-Inducing Cyanobacterium *Nostoc cycadae* Strain WK-1, Isolated from the Coralloid Roots of *Cycas revoluta*

**DOI:** 10.1128/genomeA.00021-18

**Published:** 2018-02-15

**Authors:** Yu Kanesaki, Masaki Hirose, Yuu Hirose, Takatomo Fujisawa, Yasukazu Nakamura, Satoru Watanabe, Shigeru Matsunaga, Hiroko Uchida, Akio Murakami

**Affiliations:** a NODAI Genome Research Center, Tokyo University of Agriculture, Tokyo, Japan; b Research Institute of Green Science and Technology, Shizuoka University, Shizuoka, Japan; c Department of Biology, Faculty of Education, Wakayama University, Wakayama, Japan; d Department of Environmental and Life Sciences, Toyohashi University of Technology, Toyohashi, Aichi, Japan; e Center for Information Biology, National Institute of Genetics, Research Organization of Information and Systems, Mishima, Shizuoka, Japan; f Department of Bioscience, Tokyo University of Agriculture, Setagaya, Tokyo, Japan; g Hayama Center for Advanced Studies, Graduate University for Advanced Studies, Hayama, Kanagawa, Japan; h Kobe University Research Center for Inland Seas, Awaji, Hyogo, Japan

## Abstract

We report here the whole-genome sequence of *Nostoc cycadae* strain WK-1, which was isolated from cyanobacterial colonies growing in the coralloid roots of the gymnosperm *Cycas revoluta*. It can provide valuable resources to study the mutualistic relationships and the syntrophic metabolisms between the cyanobacterial symbiont and the host plant, *C. revoluta*.

## GENOME ANNOUNCEMENT

Sago palm (*sotetsu* in Japanese; *Cycas revoluta*) is an evergreen woody plant of the family Cycadaceae in the class Gymnospermae and is distributed naturally around the Nansei Islands and southern Kyushu in Japan. The slow but perpetual growth of *C. revoluta* under sterile habitats, such as the wasteland of gravelly substratum along the seashore, is supported by the heterocyst-forming and nitrogen-fixing cyanobacterial symbionts. These cells are colonized regularly and densely in a specialized underground organ (coralloid root), which is a dichotomously branching and apogeotropic secondary root (1). The cyanobacteria *in hospite* are rich in heterocysts and mostly light in color, owing to a small amount of chlorophyll *a* and phycocyanin. The cyanobacterial strain *Nostoc cycadae* WK-1 (renamed from WUEB-01) was isolated by M. Hirose from the coralloid root of *C. revoluta* planted on the campus of Wakayama University, Wakayama, Japan, in 1977 (2, 3). The cyanobacterium typically differentiates elongate filaments termed hormogonia from vegetative filamentous cells. The hormogonia exhibit temporally the chemotactic and/or phototactic gliding motility involved in the infection processes toward the host plant tissue (1). *N. cycadae* strain WK-1 keeps the potential of cell differentiation and can be cultured at a high density on an agar plate or in liquid medium using a modified Detmer’s medium (4) or BG-11 medium (5).

The isolation of cyanobacterial genomic DNA (gDNA) and whole-genome sequencing were performed using a MiSeq sequencer (Illumina, San Diego, CA) with the MiSeq reagent kit version 3 (600 cycles; Illumina), as shown in previous reports (6–8). An 800-bp paired-end library and an 8-kbp mate pair library were prepared using the TruSeq DNA PCR-free sample preparation kit (Illumina) and Nextera mate pair sample preparation kit (Illumina), respectively. The obtained reads were cleaned by removing low-quality (<25 Phred score) and short (<100-bp) reads. A total of 794 Mbp of paired-end reads were assembled using CLC Genomics Workbench version 7.5 (Qiagen), yielding 252 scaffolds (>1 kbp). A number of scaffolds obviously derived from contaminated bacteria were removed by manual curation using a BLAST search. Finally, we obtained 68 cyanobacterium-derived scaffolds with high similarity to the genus *Nostoc*. Gene annotation was performed by MiGAP (9) and by manual curation referring to CyanoBase (10, 11).

The total length of the draft genome sequence of *N. cycadae* strain WK-1 without gap regions was 6,773,844 bp, with a G+C content of 39.06%. Of the total predicted genes, 5,465 coding sequences (CDSs), 3 rRNAs, and 71 tRNAs were identified. The 16S rRNA sequence of *N. cycadae* WK-1 showed the highest similarity (99.6%) with that of the most closely related species, *Nostoc* sp. strain PCC 7107, which is a free-living isolate from the shallow pond and whose complete genome sequence has been deciphered (12). The genome size and the number of CDSs in strain WK-1 are slightly larger than those of PCC 7107 (6.33 Mb and 5,317 CDSs). Further comparison of these species will largely contribute to an understanding of the unique and symbiotic relationship between nitrogen-fixing cyanobacteria and the gymnosperm *Cycas revoluta*.

### Accession number(s).

The sequences and annotations of the 68 scaffolds of *Nostoc cycadae* WK-1 have been deposited in DDBJ/ENA/GenBank under the accession number BDGE00000000. The version described in this paper is the first version.
